# Development and validation of a prediction algorithm to identify birth in countries with high tuberculosis incidence in two large California health systems

**DOI:** 10.1371/journal.pone.0273363

**Published:** 2022-08-25

**Authors:** Heidi Fischer, Lei Qian, Jacek Skarbinski, Katia J. Bruxvoort, Rong Wei, Kris Li, Laura B. Amsden, Mariah S. Wood, Abigail Eaton, Brigitte C. Spence, Sally F. Shaw, Sara Y. Tartof

**Affiliations:** 1 Department of Research and Evaluation, Kaiser Permanente Southern California, Pasadena, California, United States of America; 2 Division of Research, Kaiser Permanente Northern California, Oakland, California, United States of America; 3 Department of Infectious Diseases, Oakland Medical Center, Kaiser Permanente Northern California, Oakland, California, United States of America; 4 Department of Epidemiology, University of Alabama at Birmingham, Birmingham, Alabama, United States of America; 5 Department of Health Systems Science, Kaiser Permanente Bernard J. Tyson School of Medicine, Pasadena, California, United States of America; Vietnam National University, VIET NAM

## Abstract

**Objective:**

Though targeted testing for latent tuberculosis infection (“LTBI”) for persons born in countries with high tuberculosis incidence (“HTBIC”) is recommended in health care settings, this information is not routinely recorded in the electronic health record (“EHR”). We develop and validate a prediction model for birth in a HTBIC using EHR data.

**Materials and methods:**

In a cohort of patients within Kaiser Permanente Southern California (“KPSC”) and Kaiser Permanent Northern California (“KPNC”) between January 1, 2008 and December 31, 2019, KPSC was used as the development dataset and KPNC was used for external validation using logistic regression. Model performance was evaluated using area under the receiver operator curve (“AUCROC”) and area under the precision and recall curve (“AUPRC”). We explored various cut-points to improve screening for LTBI.

**Results:**

KPSC had 73% and KPNC had 54% of patients missing country-of-birth information in the EHR, leaving 2,036,400 and 2,880,570 patients with EHR-documented country-of-birth at KPSC and KPNC, respectively. The final model had an AUCROC of 0.85 and 0.87 on internal and external validation datasets, respectively. It had an AUPRC of 0.69 and 0.64 (compared to a baseline HTBIC-birth prevalence of 0.24 at KPSC and 0.19 at KPNC) on internal and external validation datasets, respectively. The cut-points explored resulted in a number needed to screen from 7.1–8.5 persons/positive LTBI diagnosis, compared to 4.2 and 16.8 persons/positive LTBI diagnosis from EHR-documented birth in a HTBIC and current screening criteria, respectively.

**Discussion:**

Using logistic regression with EHR data, we developed a simple yet useful model to predict birth in a HTBIC which decreased the number needed to screen compared to current LTBI screening criteria.

**Conclusion:**

Our model improves the ability to screen for LTBI in health care settings based on birth in a HTBIC.

## Introduction

Active tuberculosis (TB) disease causes substantial morbidity and mortality. *Mycobacterium tuberculosis* infection is spread from person to person through the air. Most persons exposed to *M*. *tuberculosis* will develop latent tuberculosis infection (LTBI), an asymptomatic infection, but have an estimated lifetime risk of 5 to 10% of developing active TB disease from reactivation of their LTBI [1]. Treatment of persons with LTBI with a short course of antituberculosis medications is very effective at reducing the risk of reactivation and a key strategy in reducing the overall burden of active TB disease in the world. An estimated 25% of the world population has LTBI with the vast majority residing outside the United States [2].

In 2020, 71% of TB cases in the US occurred among non-US-born persons [3]. An estimated 90% of TB cases in non-US born persons in the United States are attributable to reactivation of LTBI. Therefore, targeted testing for and treatment of LTBI among persons born in higher TB incidence countries is an effective strategy to decrease TB incidence in the US [4]. This could be especially true in California, home to more than 10 million non-U.S.-born persons, vastly more than any other state in the United States. Twenty-seven percent of California’s population is born outside the U.S., approximately twice the U.S. percentage [5]. In 2020, 84% of active TB cases in California occurred in non-US-born persons [6].

LTBI screening in persons born in high TB incidence countries (“HTBIC”) has historically been implemented through the public health sector and focused on those newly arrived to the US [7–9]. However, in recent years, more US TB diagnoses among persons born in HTBIC occurred ≥10 years after arrival in the U.S. than among those in the US <10 years [10]. As such, reactivation of LTBI remains a concern in persons born in HTBICs who have lived in the United States for many years. Effective LTBI screening in this population requires moving beyond the public health sector to expand partnerships with both private and public health care providers [4, 10, 11]. Risk assessment tools developed by the California Department of Public Health (CDPH) and the US Preventative Service Task Force were developed in 2016 and strongly suggest prioritizing the screening persons born in HTBIC [12, 13].

Unfortunately, country of birth is not routinely recorded in the electronic health record (EHR). With health systems and providers struggling under the burden of multiple conflicting priorities and new demands brought about by the pandemic, it is currently not feasible to enact a comprehensive system to reach out to patients collect this information or to require physicians to ask this question during office visits for millions of patients. As such, this information is often missing for a large percentage of patients, greatly diminishing the effectiveness of the LTBI screening guidelines.

In the absence of information on country of birth, information on preferred spoken language, already routinely collected in most medical records, has been used as a proxy to determine country of birth. However, with many non-US-born persons preferring to use English when seeking medical care, this indicator may greatly under-detect the true number of persons born in HTBICs [14]. As such, we sought to develop and validate a diagnostic multivariable prediction model for use in the primary care setting in California that uses variables available in the EHR to predict birth in a HTBIC. We explored various cut-points to predict birth in a HTBIC and evaluated the algorithm’s usefulness for screening both LTBI and TB. This model could be used to efficiently prompt providers to consider LTBI screening during primary care visits, or as part of a larger, targeted external outreach program in health care organizations. While this algorithm has direct benefit for LTBI screening, it may also be relevant for screening of other diseases with higher burdens in patients born in HTBICs.

## Methods

### Source of data

We aimed to predict birth in a HTBIC in a cohort of patients within Kaiser Permanente Southern California (KPSC) and Kaiser Permanente Northern California (KPNC) between January 1, 2008 and December 31, 2019. KPSC and KPNC are large, integrated health systems serving over 9.2 million racially and socioeconomically diverse members in 489 medical offices [15, 16]. Approximately 27.2% and 46.5% of patients at KPSC and KPNC, respectively, had information on country of birth in the EHR. The KPSC population was used as the development dataset, while the KPNC dataset was used for external validation.

### Ethics

The KPSC Institutional review board approved this study and granted a waiver of informed consent, as the data-only research activities were determined to pose minimal risk.

### Study population

Patients were required to be 18 years or older at some point during the study period and to have at least one year of continuous membership with a 45-day enrollment gap allowed at either KPSC or KPNC.

### Outcome

The outcome of interest was birth in a HTBIC, using the definition defined for adult LTBI screening by the CDPH [17]. Specifically, using information on country of birth available in the EHR, all patients born outside of the United States, northern or western Europe, Canada, or Australia/New Zealand were determined to be born in a HTBIC.

### Predictors

Predictors for birth in a HTBIC were determined through input from clinicians and subject matter experts. Individual level predictors came from the EHR, and included whether the patient indicated that they preferred to speak a language primarily spoken in a HTBIC per the CDPH definition (“preferred language”), whether they wanted an interpreter at office visits (“needs interpreter”), the patient’s self-reported race/ethnicity (non-Hispanic white, non-Hispanic black, Hispanic, Asian, Hawaiian/Pacific Islander, Native American/Alaskan, or Other/Multiple/Unknown), whether the patient received a Bacillus Calmette–Guérin vaccination in the past for tuberculosis (“BCG vaccination”), and whether the patient was screened at any point for hepatitis B (“screened for HBV”).

As an area-level predictor, we included the percent non-US born population living in patient’s residential census tract as reported by the 2019 American Community Survey (“percent non-US born in census tract”). The vast majority of non-US-born persons in California are born in HTBICs according to our definition [5].

Due to the larger number of patients missing race/ethnicity who were also missing country of birth in the EHR, those missing race/ethnicity were categorized into the Other/Multiple/Unknown categories. There was no imputation of missing data for other predictors where missingness was rare. Patients with these missing predictors were not included in model development; however, once the final model was chosen, predictive performance for sub-models estimated using the predictors available for each patient was evaluated to understand if useful screening recommendations could be made for these patients.

Detailed definitions and rational for inclusion of these predictors are provided in S1 Appendix.

### Statistical analysis

The KPSC dataset was split into training and testing components in a 75/25 split. The relationships between predictors and outcomes, as well as selection of final model predictors and estimates for parameter values were assessed using only the training dataset at KPSC. Once a final model was chosen and fit, performance was evaluated using the KPSC test data (internal validation), and then the KPNC data (external validation).

To maximize interpretability and facilitate implementation in the clinical setting, we derived a prediction model using logistic regression. Categorical variables were entered into the models in categories specified above, while percent non-US born in census tract was included in models as a continuous variable.

We assessed model performance by evaluating the following 3 metrics on the KPSC training dataset: area under the receiver operator curve (AUCROC), Brier score, and the area under the precision (PPV) and recall (sensitivity) curve (AUPRC) [18]. AUCROC and AUPRC were jointly considered as primary metrics. Random performance for AUCROC is indicated by a score of 0.5. Random performance for AUPRC is equal to sample prevalence of the outcome of interest, with scores higher than the sample prevalence (up to a score of 1) indicating superior model performance. The Brier score is equivalent to mean squared error as applied to predicted probabilities, meaning lower values are preferred. Ninety-five percent confidence intervals (95% CIs) are presented for all three metrics [19–21]. To further evaluate model calibration, we examined model calibration plots, which were loess plots of predicted vs actual probabilities for the final model on the training, test, and external validation datasets [22]. Model performance was evaluated for each predictor individually, in a fully-adjusted model including all two-way interactions, in a model including only main-effects, then iteratively removing predictors in a stepwise fashion. To satisfy our goal to use the simplest, intuitive model possible with satisfactory predictive power, predictors were iteratively removed in an order reflecting both their predictive and clinical values. The model building process is explained in detail in S2 Appendix. Once the final model was chosen, it was internally validated using the KPSC test dataset and then externally validated using the KPNC dataset, where predicted probabilities were calculated using the logit of the sum of logistic regression coefficients estimated on the training dataset.

Using the final model, we explored various cut-points by calculating the proportion with a positive result with that cut-point for which the true condition is positive (Positive Predictive Value, PPV), proportion of the individuals for which the true condition is positive receiving a negative result (False Omission Rate, FOR), sensitivity, and specificity using the internal and external validation datasets. Cut-points explored were chosen so that the number screened would match the estimated percentage (based on non-missing country of birth data in the EHR) of members born in HTBICs at KPNC (19%), KPSC (24%), as well as the percent non-US-born in California (27%) and Los Angeles County (34%) [5]. Results using these cut-points were further compared to the model using language only to predict birth in HTBIC since language is a commonly used predictor in the absence of this information [14].

To better understand how this model might be directly useful to LTBI screening, we also applied these cut-points to screen patients with positive LTBI and TB diagnoses. LTBI diagnoses were made using tuberculin skin tests (TST) or interferon gamma release assays (IGRA), while active TB was diagnosed using polymerase chain reaction (PCR) and culture methods. Since patients with LTBI diagnoses do not have symptoms and only a portion of patients are currently screened in KPSC and KPNC health systems, we were only able to evaluate the models’ use for LTBI for those screened for LTBI at any point in the study period. As TB is symptomatic, we evaluated TB screening for the full population, regardless of LTBI screening. We examined the number screened, true positive rate (LTBI or TB diagnoses identified for each cut-point divided by total LTBI or TB diagnoses), absolute number of LTBI or TB diagnoses captured for each cut-point, and the number needed to screen (NNS, defined as the number screened divided by the number of positive diagnoses for each cut-point) to explore how various cut-points might capture LTBI and TB diagnoses and minimize screening. This was done in the internal and external validation datasets, as well as for patients missing information on country of birth in the EHR.

The study adhered to the TRIPOD (Transparent Reporting of a multivariable prediction model for Individual Prognosis Or Diagnosis) statement for reporting [23]. All models were developed by using the R software version 4.0.4 (R Foundation for Statistical Computing, Vienna, Austria) [24].

## Results

### Participants

The KPSC cohort had 7,482,417 people, of which 496,257 (6.6%) had documentation in the EHR of birth in HTBIC, 1,540,143 (20.6%) had documentation of showing they were not born in a HTBIC, and 5,446,017 (72.8%) were missing information on country of birth in the EHR (Table 1). EHR documented patients born in HTBICs were much more likely to prefer to speak a language spoken in a country with high TB incidence and need an interpreter, 37% and 30% respectively, versus those who were not born in a HTBIC, 2.2% and 1.5%, respectively. Patients with EHR documented birth in HTBICs were more likely to be Asian, Hispanic, or Hawaiian/Pacific Islanders, compared to those not born in a HTBIC, who were more likely to be White, Black, or Native American/Alaskan. Patients with EHR documented birth in HTBICs tended to live in neighborhoods with a higher percentage of non-US-born residents compared to those who were not born in a HTBIC (a median of 34 vs 26 percent), and slightly more were screened for hepatitis B (35 vs 30 percent, respectively). A negligible number of patients recorded being previously vaccinated for BCG in the EHR regardless of EHR documentation of country of birth. Those missing country of birth information in the EHR tended to be similar to those with non-missing information, except HBV screening was lower in the missing population, and missingness was higher for all predictor variables, particularly race/ethnicity.

**Table 1:** Characteristics of KPSC patient population by country of birth, as documented in the electronic health record, January 1, 2008—December 31^st^, 2019.

Characteristic	Born in-HTBIC^1^	Not Born in HTBIC	Total	Missing
	N = 496,257	N = 1,540,143	N = 2,036,400	N = 5,446,017
**Preferred Language Spoken in HTBIC, n (%)**				
No	308,353 (62.1)	1,504,729 (97.7)	1,813,082 (89)	4,631,579 (85.0)
Yes	183,670 (37.0)	34,522 (2.2)	218,192 (10.7)	620,004 (11.4)
Unknown	4,234 (0.9)	892 (0.1)	5126 (0.3)	194434 (3.6)
**Needs Interpreter During Office Visits, n (%)**				
No	345,387 (70)	1,515,940 (98)	1,861,327 (91)	4,847,902 (89)
Yes	149,353 (30)	23,083 (1.5)	172,436 (8.5)	378,583 (7.0)
Unknown	1,517 (0.3)	1,120 (<0.1)	2,637 (0.1)	219,532 (4.0)
**Percent Non-US-Born in US Census Tract**				
Median (IQR)	34 (25, 43)	26 (17, 36)	28 (19, 38)	26 (17, 36)
Unknown, n (%)	876 (0.2)	3,268 (0.2)	4144 (0.2)	30,722 (0.6)
**Race/Ethnicity, n (%)**				
White	48,757 (9.8)	691,669 (45)	740,426 (36)	1,574,527 (29)
Asian	132,132 (27)	80,553 (5.2)	212,685 (10)	501,022 (9.2)
Black	8,987 (1.8)	203,434 (13)	212,421 (10)	351,783 (6.5)
Hawaiian/Pacific Islander	7,400 (1.5)	8,577 (0.6)	15,977 (0.8)	35,177 (0.6)
Hispanic	294,339 (59)	538,884 (35)	833,223 (41)	2,028,575 (37)
Native Am./Alaskan	658 (0.1)	4,862 (0.3)	5,520 (0.3)	14,864 (0.3)
Multiple/Other/Unknown	3,984 (0.8)	12,164 (0.8)	16,148 (0.8)	940,069 (17)
**Previously vaccinated for BCG** ^**2**^ **, n (%)**				
No	495,926 (100)	1,539,744 (100)	2,035,670 (100)	5,442,120 (100)
Yes	331 (<0.1)	399 (<0.1)	730 (<0.1)	3,897 (<0.1)
**Ever screened for HBV** ^**3**^ **, n (%)**				
No	322,862 (65)	1,078,472 (70)	1,401,334 (69)	4,690,499 (86)
Yes	173,395 (35)	461,671 (30)	635,066 (31)	755,518 (14)

^1^high TB incidence country ^2^Bacillus Calmette–Guérin vaccination, ^3^hepatitis B virus

The KPNC cohort had 6,190,227 patients, of which 559,865 (9.0%) had EHR documented births in HTBICs, 2,320,705 (37.5%) had documentation showing they were not born in a HTBIC, and 3,309,657 (53.5%) were missing information on country of birth in the EHR. Patterns between categories were similar to KPSC (S1 Table). The patient populations for KPSC and KPNC were largely similar on the predictors of interest (S2 Table), except KPSC had a higher proportion of patients identifying as Hispanic (38% for KPSC vs 21% for KPNC), while KPNC had a higher proportion of patients identifying as Asian (18% for KPNC vs 9.5% for KPSC). Additionally, 24% of patients with information on country of birth in the EHR were born in a HTBIC, while 19% of patients with information on country of birth in the EHR were born in a HTBIC at KPNC.

### Model development, specification, and performance

**Fig 1:**
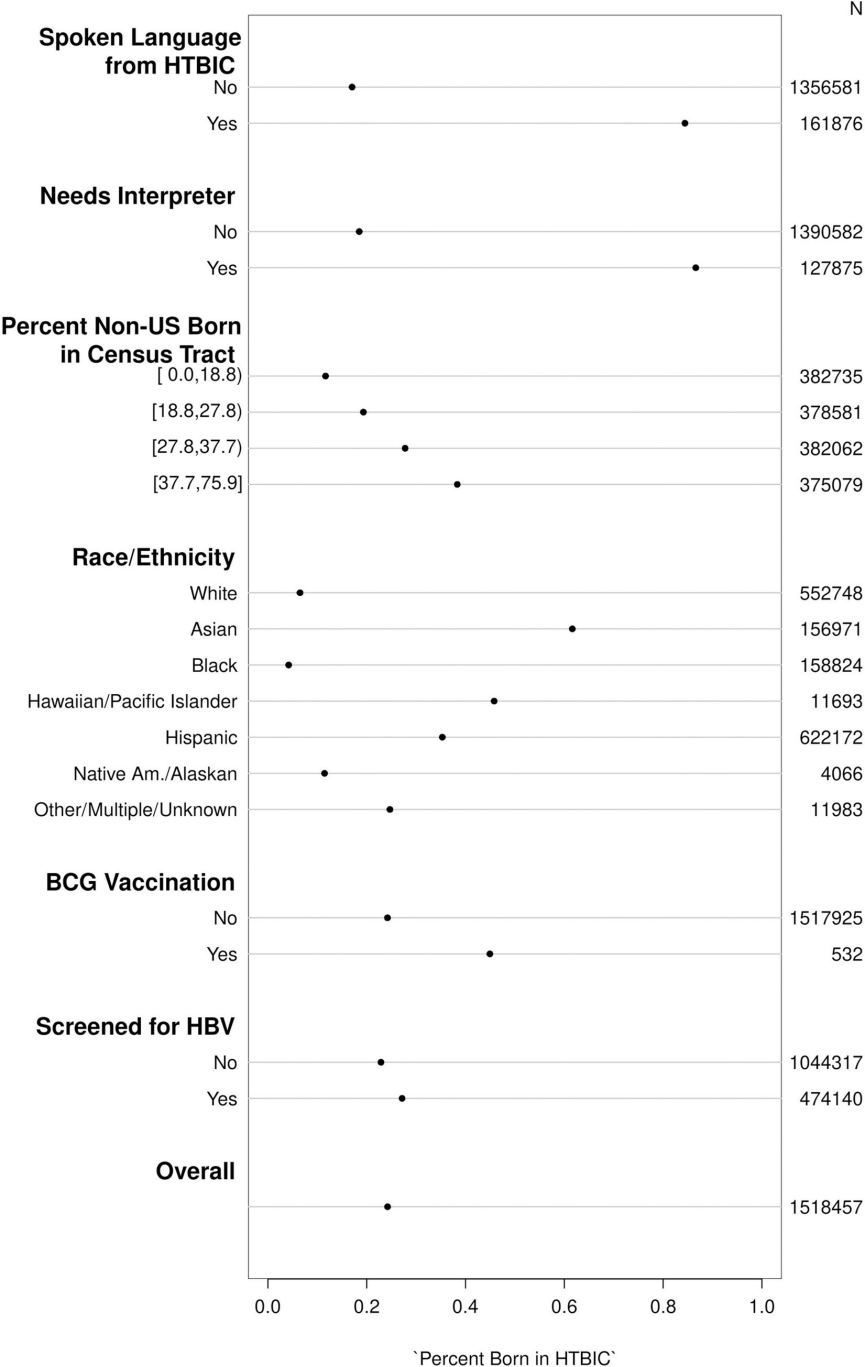
Percent patients born in countries with high TB incidence for each predictor of interest in the training dataset.

Of the 7,482,417 patients in the KPSC cohort, 2,036,400 (27.2%) had information on country of birth. An additional 11,791 (0.6%) were excluded for having incomplete covariate information, leaving 2,024,609 patients in the dataset for prediction. This was split further into a 75/25 training/test split for model development. Fig 1 summarizes the percent of patients born in HTBICs for each predictor of interest in the training dataset, along with giving the absolute number of events. Visual inspection of loess curves showed a linear relationship between birth in HTBIC and the probability non-US-born in patient’s census tract, so this variable was included as a linear term in predictive models. Fig 1 demonstrates that preferred language, need for interpreter during office visits, and race/ethnicity are the strongest univariate predictors.

Table 2 contains model performance metrics for all models considered. Performance metrics for two-way interactions and main effects were similar, both achieving an AUCROC of 0.86 (95% CI 0.86–0.86) and a AUPRC of 0.71 (95% CI 0.71–0.71) and 0.72 (95% CI 0.72–0.72), respectively (compared to a baseline HTBIC-birth prevalence of 0.24). This indicated excellent predictive value in a model containing only the main effects, and that a more complicated model including interactions was not necessary. Predictors were then iteratively removed from the model in an order reflecting both their predictive and clinical values to explore if further simplification was possible. This process is explained in detail in S2 Appendix. Our final model containing preferred language, race/ethnicity, and percent non-US-born in patient’s census tract had an AUCROC 0.85 (95% CI 0.85–0.85), an AUPRC of .70 (95% CI 0.70–0.70), and a Brier score of 0.12 (95% CI 0.12–0.12).

**Table 2:** Model performance with 95% confidence intervals.

Model	AUCROC^1^	AUPRC^2^	Brier
Models on Training Dataset			
Preferred Language	0.68 (0.67,0.68)	0.53 (0.53,0.54)	
Needs Interpreter	0.64 (0.64,0.64)	0.50 (0.50,0.50)	
Percent Non-US-Born in Census Tract	0.66 (0.66,0.66)	0.37 (0.37,0.37)	
Race Ethnicity	0.77 (0.77,0.77)	0.49 (0.48,0.49)	
BCG^3^ Vaccine	0.50 (0.50,0.50)	0.24 (0.24,0.24)	
HBV^4^ Screen	0.52 (0.52,0.53)	0.26 (0.26,0.26)	
Two-Way Interactions	0.86 (0.86,0.86)	0.72 (0.72,0.72)	0.11 (0.11,0.11)
Main Effects	0.86 (0.86,0.86)	0.71 (0.71,0.71)	0.12 (0.11,0.12)
Language, Race/Ethnicity, Census, Interpreter, HBV	0.86 (0.86,0.86)	0.71 (0.71,0.71)	0.12 (0.12,0.12)
Language, Race/Ethnicity, Census, Interpreter	0.86 (0.85,0.86)	0.70 (0.70,0.70)	0.12 (0.12,0.12)
**Final Model: Language, Race/Ethnicity, Non-US-born in Census Tract**	**0.85 (0.85,0.85)**	**0.70 (0.70,0.70)**	**0.12 (0.12,0.12)**
Language, Race/Ethnicity	0.84 (0.84,0.84)	0.68 (0.68,0.68)	0.12 (0.12,0.12)
**Final Model on Test Dataset: Language, Race/Ethnicity, Non-US-Born in Census Tract**	**0.85 (0.85,0.86)**	**0.69 (0.69,0.70)**	**0.12 (0.12,0.12)**
**KPNC External Validation: Language, Race/Ethnicity, Non-US-Born in Census Tract**	**0.87 (0.87,0.87)**	**0.64 (0.64, 0.64)**	**0.10 (0.10,0.10)**

^1^area under the receiver operator curve

^2^area under the precision and recall curve

^3^Bacillus Calmette–Guérin vaccination

^4^Hepatitis B

**Fig 2:**
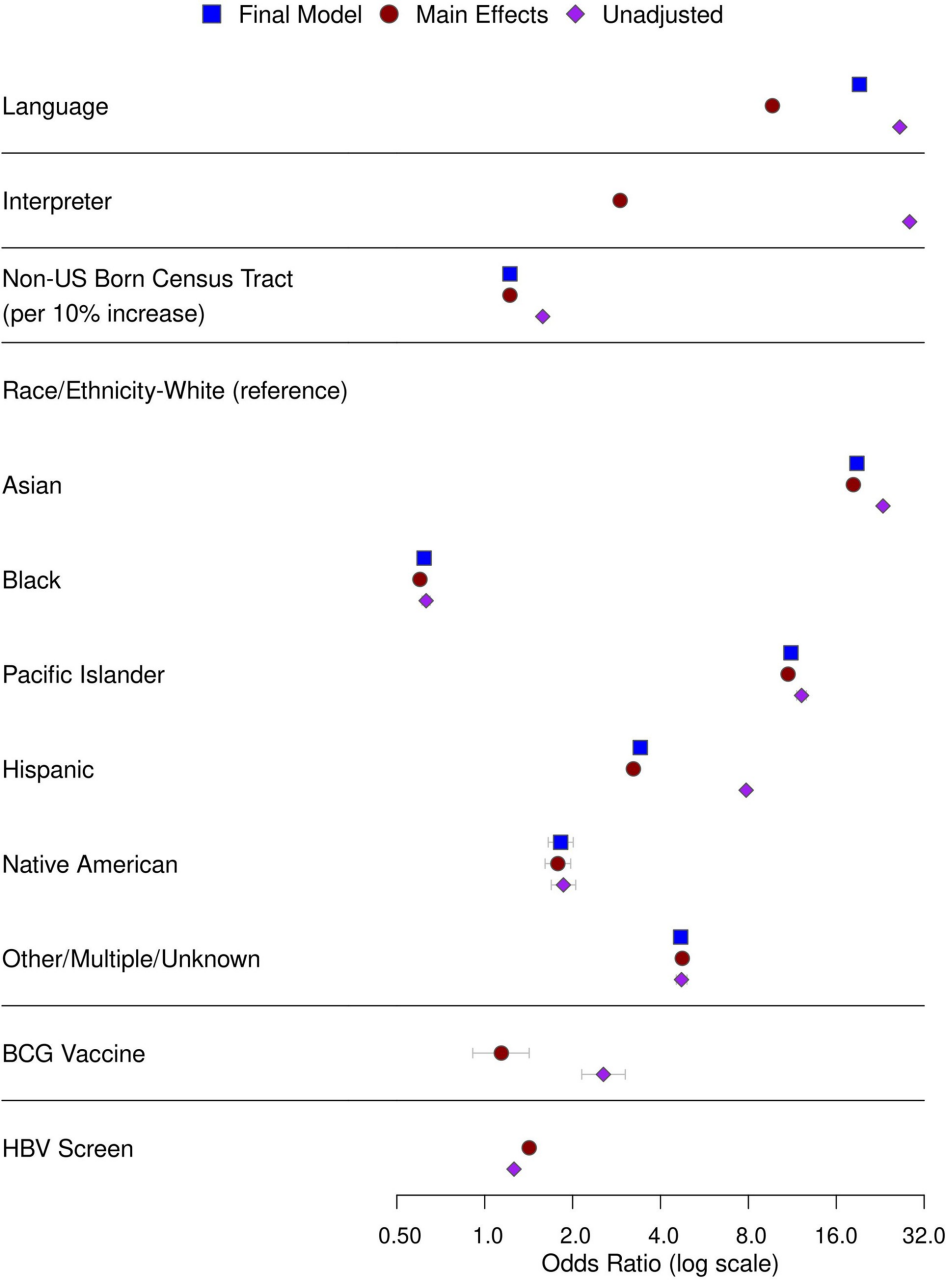
Odds ratios and 95 percent confidence intervals for predictors in univariate, main effects, and final models.

Fig 2 compares odds ratios (ORs) from the unadjusted, fully adjusted main effects, and final chosen models. Exact model estimates are provided in S3 Table and the full prediction model is presented in S4 Table. Performance metrics on the internal KPSC test and external KPNC validation datasets for the final model were comparable to those in the training dataset, with an AUC of 0.87 (95% CI 0.87–0.87) and an AUPRC of 0.64 (95% CI 0.64–0.64, compared to a baseline HTBIC-birth prevalence of 0.19 at KPNC) on the external KPNC validation dataset. However, the model tended to over-predict birth in HTBIC for probabilities over 0.2 for the external KPNC validation dataset (Table 2, S1 Fig). For KPSC and KPNC patients who were missing predictors included in the final model, predictive performance for sub-models estimated using the predictors available for each patient showed comparable AUC and AUPRC, with slightly higher Brier scores (S5 Table).

### Cut-points and application to LTBI and TB screening

Cut-points were chosen using population percentages in the training dataset. Table 3 shows a sensitivity of 0.37 and 0.29 and a PPV of 0.84 and 0.77 for the language only models on the internal KPSC and external KPNC validation datasets, respectively. In comparison, the final model with the most inclusive cut-point (screening 34% of the population) resulted in true-positive rates of as much as 0.74 and 0.78 for KPSC and KPNC respectively, with a trade-off in PPV to be as low as 0.53 and 0.51, respectively. A similar relationship was observed between the true-negative rate and the False Omission Rate, FOR.

**Table 3:** Final model performance on validation datasets using various cut-points.

Model	PPV^1^	FOR^2^	Sensitivity	Specificity
**KPSC Internal Validation**	** **	** **	** **	** **
Preferred Language Only	0.84	0.17	0.37	0.98
19% Cut-point (KPNC estimate)	0.74	0.13	0.58	0.93
24% Cut-point (KPSC Estimate)	0.66	0.11	0.65	0.89
27% Cut-point (CA^3^ Estimate)	0.61	0.11	0.68	0.86
34% Cut-point (LA^4^ County Estimate)	0.53	0.09	0.74	0.79
**KPNC External Validation**				
Preferred Language Only	0.77	0.15	0.29	0.98
19% Cut-point (KPNC estimate)	0.61	0.09	0.64	0.9
24% Cut-point (KPSC Estimate)	0.56	0.07	0.73	0.86
27% Cut-point (CA Estimate)	0.54	0.07	0.75	0.85
34% Cut-point (LA County Estimate)	0.51	0.06	0.78	0.82

^1^Positive predictive value

^2^False Omission Rate, ^3^California, ^4^Los Angeles

Table 4 applies these same cut-points to show how the model performed for LTBI and TB screening in patients with known country of birth at KPSC and KPNC; the population for the LTBI diagnosis endpoint includes only those screened for LTBI in the KPSC/KPNC cohorts while the population for the TB endpoint includes all patients. For both LTBI and TB endpoints, the table displays the number of patients that would be screened using each cut-point, number of diagnoses identified among screened patients, true positive rate, and the NNS. The final model using any of the above cut-points improved the true positive rate for both LTBI and TB by over 30 percentage points compared to a language-only model. Models using the most conservative cut-point, screening 19% of the population, identified an additional 14,000 and 640 additional positive LTBI and TB diagnoses, respectively, compared to using a language only model. The 19% cut-point resulted in a 0.49 and 0.69 true positive rate, while the 34% cut-point resulted in a .63 and .81 true positive rate, compared to a 0.66 and 0.78 true positive rate for EHR-documented birth in HTBIC, for LTBI and TB, respectively.

**Table 4:** Screening metrics for LTBI and TB in patients with known country of birth for final model using various cut-points.

Model Combination	N Screened	True Cases Identified	True Positive Rate	NNS^1^
**LTBI**^2^ **(applied to population screened for LTBI, N = 802,209)**				
Preferred Language Only	50,034	8601	0.18	5.8
Final Model, 19% Cut-point	163,321	23129	0.49	7.1
Final Model, 24% Cut-point	208,306	27142	0.57	7.7
Final Model, 27% Cut-point	223,607	28048	0.59	8.0
Final Model, 34% Cut-point	254,845	29884	0.63	8.5
EHR Documented Birth in HTBIC	132,125	31507	0.66	4.2
**TB**^3^ **(applied to full population, N = 3,361,893) **				
Preferred Language Only	264,731	291	0.38	909.7
Final Model, 19% Cut-point	671,991	931	0.69	721.8
Final Model, 24% Cut-point	844,211	1045	0.77	807.9
Final Model, 27% Cut-point	898,033	1059	0.78	848.0
Final Model, 34% Cut-point	1,011,946	1091	0.81	927.5
EHR Documented Birth in HTBIC	673,742	1050	0.78	641.7

^1^Number needed to screen defined as number screened divided by number of cases identified

^2^latent tuberculosis infection

^3^active tuberculosis disease

For LTBI, the NNS for selected cut-points ranged from 7.1–8.5 persons/positive LTBI diagnosis, compared to 4.2 for EHR-documented birth in HTBIC and 5.8 for persons/positive LTBI diagnosis for language. Using any of these cut-points would lead to an improvement from the NNS of 16.8 persons/positive LTBI case which has resulted from current LTBI screening criteria at these health systems. For active TB, the cut-point which screens 19% of the population achieved the NNS closest to EHR-documented birth in HTBIC (721.8 persons/positive TB case vs. 641.7 persons/positive TB case). Among those without information on country of birth at both KPSC and KPNC, the final model using these cut-points showed similar true-positive rates and patterns for NNS compared with the language only model, thought the NNS was higher across for all metrics for the active TB outcome. (S6 Table).

## Discussion

Using a logistic regression model with only 3 variables available from the patient’s EHR, we were able to predict birth in a HTBIC with an AUCROC over 0.85 on both internal and external validation datasets and an AUPRC of 0.69 (compared to a baseline prevalence of 0.24) and 0.64 (compared to a baseline prevalence of 0.19) for internal and external validation datasets, respectively. While a model using language only had very high PPV compared to our final model using pre-specified cut-points, the true positive rate was only 0.37 and 0.29 in internal and external validation datasets, respectively. Although language preference is an accurate predictor of birth in a HTBIC when a patient prefers to speak a language other than English, it misses a great deal of individuals born in HTBICs who prefer to use English when receiving medical care. The true positive rate of our final model reaches about 0.65 for both internal and external datasets when using a cut-point that would screen a number similar to the estimated percentage of those born in HTBIC at both KP sites, close to twice the true positive rate when using language alone.

Importantly, when using predictions for birth in HTBIC from the final model to screen for positive LTBI or TB, the true positive rate was similar to using EHR-documented birth in a HTBIC, even using the most conservative cut-point explored. Choosing less conservative cut-points brought the true positive even closer to that of EHR-documented birth in HTBC, with the sacrifice of more patients screened. However, while the NNS was lower for EHR-documented birth in HTBICs than all cut-points for both these endpoints, they were comparable in magnitude. The NNS estimates using the selected cut-points on those with information on country of birth ranged from 7.1–8.5 persons/positive LTBI diagnosis, all an improvement from the current NNS of 16.8 persons/positive LTBI diagnosis which has resulted from current LTBI screening criteria at these institutions; similar results were observed using the algorithm to screen those missing information on country of birth in the EHR.

The improvement in performance metrics seemed smaller after moving beyond a cut-point that would result in screening more than 24% of the population. Targeting a cut-point which may screen around 24% of the patient population, close to the estimated percentage of non-US-born residents in California, may be a good default rule of thumb in an urban Californian setting. However, it is important to note that the selection of optimal cut-points with application to LTBI or TB screening will depend on resources and priorities for case capture at each institution. The current study did not take directly take into account health effects nor costs related to screening for LTBI or acquiring active TB in evaluating various cut-points. Studies directly incorporating these inputs, which may vary by institution, may reach different conclusions. Relatedly, receiving a positive LTBI and TB diagnosis is a relatively rare event, and though birth in a HTBIC is a strong predictor, the overwhelming majority of persons in California born in HTBICs do not have LTBI, and even fewer have LTBI which will progress to active TB. Thus, it is to be expected that screening all patients that any model predicts are born in HTBICs could result in exceeding desired screening resources in some health systems, even in the event of perfect prediction. Future predictive models incorporating predicted birth in HTBIC along with other elements that may predict TB activation (immunosuppression, etc) may improve the predictive power and reduce the number needed to be screened overall. Even testing only 25% of eligible patients in primary care encounters could prevent a substantial amount of TB cases [25].

Though performance metrics such as AUCROC, AUPRC, and Brier were similar between the internal and external validation datasets, calibration plots for KPNC data showed the model tended to overpredict birth in HTBIC. However, using the pre-established cut-points, the overprediction did not seem to be problematic and resulted in similar performance metrics for birth in a HTBIC as KPSC. This model could easily be implemented for use at other health-care centers in California, particularly those serving more diverse, urban dwelling patients such as those at KPSC and KPNC. Further validation should be explored if this will be used outside of California, or in non-urban areas.

This study has several limitations. Though missing race/ethnicity was included as a separate category in prediction, patients missing information on preferred language or census tract were excluded from predictions for birth in HTBIC in the final model. For those missing information on country of birth, 5.2% were missing information on language preference or census tract information at KPNC, while 5.6% were missing this information at KPSC. However, patients who were missing this information can have birth in a HTBIC predicted using alternative models including available variables only (such as only race/ethnicity and language). Performance metrices showed these sub-models still showed satisfactory predictive performance for patients missing information on preferred language and/or census tract. A strength of this model is strong predictive ability, using very few predictors, which make missing data less of an issue than more complicated models requiring an extensive number of covariates which collectively could make the missing rate quite high.

Additionally, those missing information on country of birth in the medical record may be systematically different than those who have this information in the EHR. However, the model performed similarly predicting active TB among patients with both complete and missing information on country of birth in the EHR, indicating the percent of the population born in HTBICs is not extremely imbalanced between the populations. Additionally, the percent of active TB cases detected by our definition ranged between 65–80% in both these populations, which is also in line with the actual percentage seen in California and the US in previous studies.

Finally, it should be noted that LTBI tests have imperfect sensitivity and specificity, and in particular TST may be more likely to give false positive results in the case of prior BCG vaccination [26]. TST made up 92 percent of LTBI tests during the study period. Given the percent born in high TB incidence countries in this population, it is likely that previous BCG vaccinations are under captured, possibly leading to a greater number of false positives for LTBI. Evaluation of LTBI metrics like number of additional cases captured or NNS should thus focus on comparisons across cut-points rather than absolute numbers.

In conclusion, the prediction algorithm developed in this manuscript improved our ability to screen for LTBI using birth in a HTBIC. The model identified thousands more positive LTBI cases compared to using language alone, and resulting sensitivities for LTBI and TB were comparable to using EHR-documented birth in a HTBIC. Using the developed model also resulted in a lower NNS compared to the NNS using current LTBI screening criteria at these institutions. This predictive algorithm could be used to efficiently prompt providers to consider LTBI screening during primary care visits, or as part of a larger, targeted external outreach program prompting patients to come in for screening. While this predictive algorithm has direct benefit for LTBI screening, it may also be relevant for screening of other diseases with higher burdens in patients born in high TB incidence countries.

## Supporting information

S1 Fig Calibration plots for final model. (DOCX)

S1 Table Characteristics of KPNC patient population by country of birth, as documented in the electronic health record, January 1, 2008—December 31^st^, 2019. (DOCX)

S2 Table Characteristics of overall KPNC and KPSC populations, as documented in the electronic health record, January 1, 2008—December 31^st^, 2019. (DOCX)

S3 Table Model odd ratios. (DOCX)

S4 Table Full prediction model. (DOCX)

S5 Table Model performance with 95% confidence intervals for all patients with missing predictors (KPSC, N = 11,791; KPNC, N = 24,829). (DOCX)

S6 Table Screening metrics for LTBI and TB in patients with unknown country of birth for final model using various cut-points. (DOCX)

S1 Appendix Detailed definitions for each considered predictor, along with pros and cons for considering each from a clinical and subject matter expert perspective. (DOCX)

S2 Appendix Description of model building process. (DOCX)
