# Supplementary material for: Development and validation of a prediction algorithm to identify birth in countries with high tuberculosis incidence in two large California health systems
Source: PLoS One. 2022 Aug 25;17(8):e0273363. doi: 10.1371/journal.pone.0273363 (PMC9409495; doi:10.1371/journal.pone.0273363)
Supplement: S1 Fig — (DOCX) [file pone.0273363.s001.docx]

**S1 Fig: Calibration Plots For Final Model**


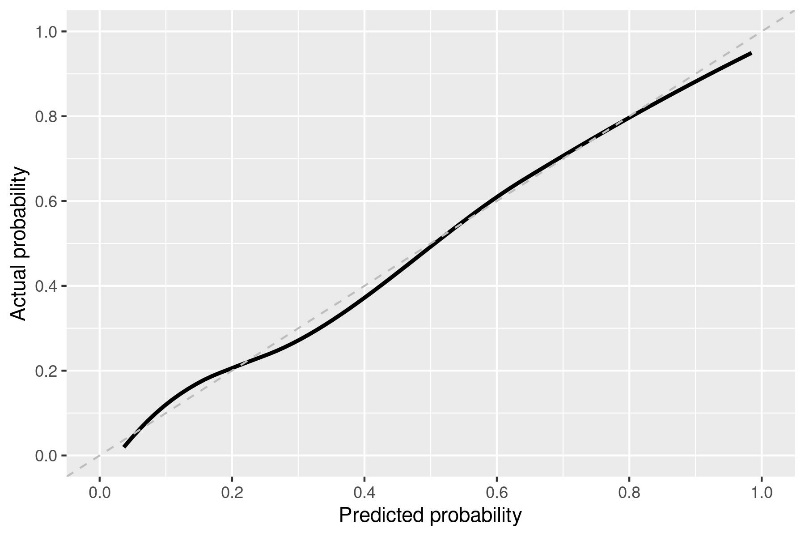


1. **KPSC Training Dataset**


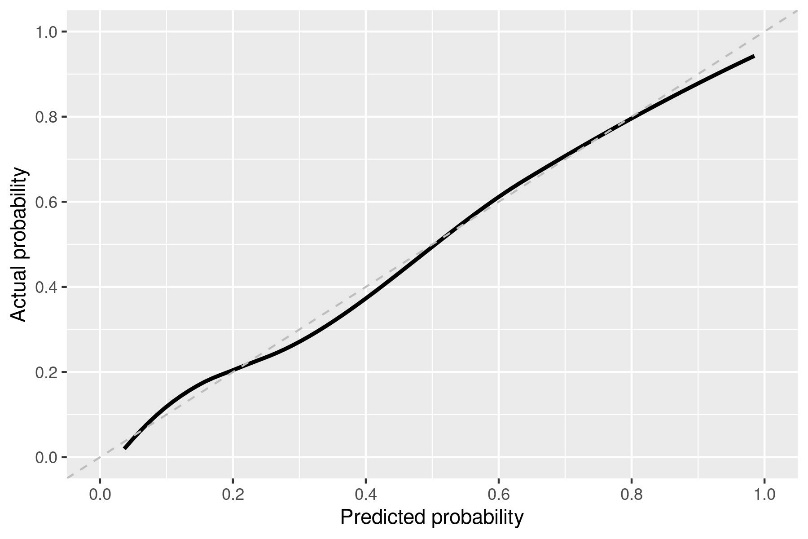


1. **KPSC Internal Validation (Test) Dataset**


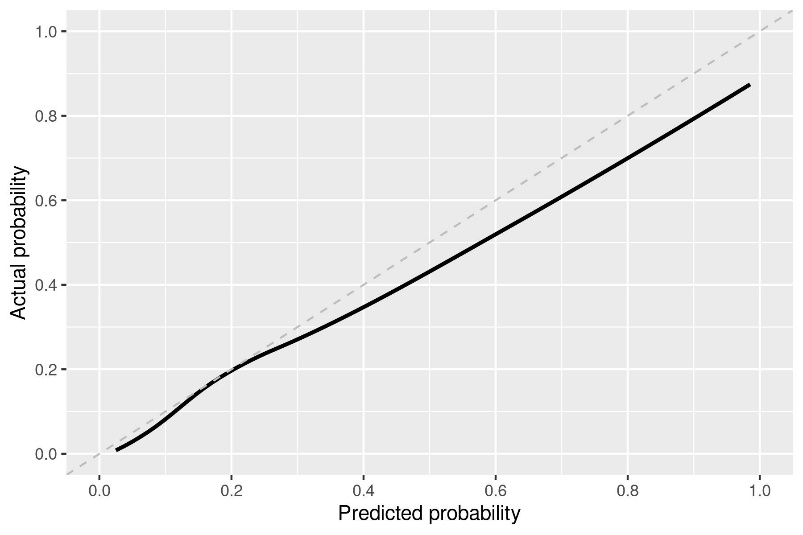


1. **KPNC External Validation Dataset**
