# Supplementary material for: Development and validation of a prediction algorithm to identify birth in countries with high tuberculosis incidence in two large California health systems
Source: PLoS One. 2022 Aug 25;17(8):e0273363. doi: 10.1371/journal.pone.0273363 (PMC9409495; doi:10.1371/journal.pone.0273363)
Supplement: S1 Table — (DOCX) [file pone.0273363.s002.docx]

**S1 Table:** **Characteristics of KPNC Patient Population by Country of Birth, as Documented in the Electronic Health Record, January 1, 2008 — December 31^st^, 2019**

| **Characteristic** | **Born in HITBC**  N = 559,865 | **Not Born in a HITBC**  N = 2,320,705 | **Missing**  N = 3,309,657 |
| --- | --- | --- | --- |
| **Preferred Language Spoken in Country with High TB Incidence** | | | |
| No | 391,705 (71) | 2,269,875 (98) | 2,847,402 (90) |
| Yes | 161,633 (29) | 49,457 (2.1) | 315,597 (10.0) |
| Unknown | 6,527 (1.2) | 1,373 (0.1) | 146,658 (4.4) |
| **Percent Foreign Born in Census Tract** | | | |
| Median (IQR) | 32 (22, 43) | 22 (14, 31) | 24 (16, 36) |
| Unknown, n (%) | 1,573 (0.3) | 15,366 (0.7) | 29,410 (0.9) |
| **Race/Ethnicity, n(%)** | | | |
| White | 51,028 (9.1) | 1,332,927 (57) | 1,249,593 (38) |
| Asian | 269,519 (48) | 218,634 (9.4) | 619,188 (19) |
| Black | 10,659 (1.9) | 239,995 (10) | 169,971 (5.1) |
| Hawaiian/Pacific Islander | 11,459 (2.0) | 18,656 (0.8) | 28,365 (0.9) |
| Hispanic | 199,917 (36) | 439,604 (19) | 637,613 (19) |
| Native Am./Alaskan | 1,038 (0.2) | 13,233 (0.6) | 15,920 (0.5) |
| Unknown (including multiple) | 16,245 (2.9) | 57,656 (2.5) | 589,007 (18) |
