# Supplementary material for: Development and validation of a prediction algorithm to identify birth in countries with high tuberculosis incidence in two large California health systems
Source: PLoS One. 2022 Aug 25;17(8):e0273363. doi: 10.1371/journal.pone.0273363 (PMC9409495; doi:10.1371/journal.pone.0273363)
Supplement: S2 Table — (DOCX) [file pone.0273363.s003.docx]

**S2 Table:** **Characteristics of Overall KPNC and KPSC Populations, as Documented in the Electronic Health Record, January 1, 2008 — December 31^st^, 2019**

| **Characteristic** | **KPSC**  N = 7,482,417 | **KPNC** N = 6,190,227 |
| --- | --- | --- |
| **Preferred Language Spoken in Country with High TB Incidence, n(%)** | | |
| No | 6,444,661 (86.1) | 5,508,982 (91) |
| Yes | 838,196 (11.2) | 526,687 (8.7) |
| Unknown | 199,560 (2.7) | 154,558 (2.5) |
| **Percent Foreign Born in US Census Tract** | |  |
| Median (IQR) | 27 (18, 36) | 24 (16, 35) |
| Unknown | 34,866 (0.5) | 46,349 (0.7) |
| **Race/Ethnicity, n (%)** |  |  |
| White | 2,314,953 (31) | 2,633,548 (43) |
| Asian | 713,707 (9.5) | 1,107,341 (18) |
| Black | 564,204 (7.5) | 420,625 (6.8) |
| Hawaiian/Pacific Islander | 51,154 (0.7) | 58,480 (0.9) |
| Hispanic | 2,861,798 (38) | 1,277,134 (21) |
| Native Am./Alaskan | 20,384 (0.3) | 30,191 (0.5) |
| Unknown (including multiple races) | 956,217 (13) | 662,908 (11) |
