# Supplementary material for: Development and validation of a prediction algorithm to identify birth in countries with high tuberculosis incidence in two large California health systems
Source: PLoS One. 2022 Aug 25;17(8):e0273363. doi: 10.1371/journal.pone.0273363 (PMC9409495; doi:10.1371/journal.pone.0273363)
Supplement: S3 Table — (DOCX) [file pone.0273363.s004.docx]

**S3 Table: Model Odd Ratios**

| **Variable** | **OR Unadjusted** | **OR Adjusted** | **OR Final Model** |
| --- | --- | --- | --- |
| **Preferred Language Spoken HTBIC** |  |  |  |
|  | 26.4 (26.03,26.78) | 9.67 (9.45,9.89) | 19.21 (18.92,19.5) |
| **Needs Interpreter** |  |  |  |
|  | 28.54 (28.07,29.02) | 2.91 (2.84,2.99) |  |
| **Percent Foreign Born in US Census Tract (per 10% increase)** |  |  |  |
|  | 1.58 (1.58,1.59) | 1.22 (1.21,1.22) | 1.22 (1.21,1.22) |
| ***Race/Ethnicity*** |  |  |  |
| White (reference) | 1.00 | 1.00 | 1.00 |
| Asian | 23.13 (22.79,23.48) | 18.31 (18.03,18.6) | 18.83 (18.55,19.12) |
| Black | 0.63 (0.61,0.65) | 0.6 (0.58,0.62) | 0.62 (0.61,0.64) |
| Hispanic | 7.86 (7.77,7.96) | 3.23 (3.19,3.27) | 3.41 (3.37,3.46) |
| Pacific Islander | 12.17 (11.71,12.64) | 10.94 (10.53,11.37) | 11.19 (10.77,11.63) |
| Native American | 1.86 (1.69,2.05) | 1.78 (1.61,1.97) | 1.82 (1.65,2.01) |
| Unknown/Multiple | 4.72 (4.53,4.93) | 4.75 (4.55,4.96) | 4.69 (4.49,4.9) |
| **BCG Vaccine** |  |  |  |
|  | 2.55 (2.15,3.03) | 1.14 (0.91,1.42) |  |
| **HBV Screen** |  |  |  |
|  | 1.26 (1.25,1.27) | 1.42 (1.4,1.43) |  |
