# Supplementary material for: Development and validation of a prediction algorithm to identify birth in countries with high tuberculosis incidence in two large California health systems
Source: PLoS One. 2022 Aug 25;17(8):e0273363. doi: 10.1371/journal.pone.0273363 (PMC9409495; doi:10.1371/journal.pone.0273363)
Supplement: S4 Table — (DOCX) [file pone.0273363.s005.docx]

**S4 Table: Full Prediction Model**

| **Variable** | **Log Odds** |
| --- | --- |
| **Intercept** | -3.2202077 |
| **Preferred Language Spoken in HTBIC** | 2.9552435 |
|  |  |
| **Percent Foreign Born in US Census Tract (per 10% increase)** |  |
|  | 0.1974384 |
| ***Race/Ethnicity*** |  |
| White (reference) | - |
| Asian | 2.9356245 |
| Black | -0.4735922 |
| Hispanic | 1.2279697 |
| Pacific Islander | 2.4150263 |
| Native American | 0.5977438 |
| Unknown/Multiple | 1.5453757 |
