# Supplementary material for: Development and validation of a prediction algorithm to identify birth in countries with high tuberculosis incidence in two large California health systems
Source: PLoS One. 2022 Aug 25;17(8):e0273363. doi: 10.1371/journal.pone.0273363 (PMC9409495; doi:10.1371/journal.pone.0273363)
Supplement: S5 Table — (DOCX) [file pone.0273363.s006.docx]

**S5 Table: Model Performance with 95% Confidence Intervals for All Patients with Missing Predictors (KPSC, N=11,791; KPNC, N=24,829).**

| **Model** | **AUCROC^1^** | **AUPRC^2^** | **Brier** |
| --- | --- | --- | --- |
| Relevant Submodel**^3^** | 0.86 (0.86,0.86) | 0.76 (0.75,0.76) | 0.15 (0.15,0.15) |

***** ^1^area under the receiver operator curve, ^2^area under the precision and recall curve, ^3^For example, patients with missing language and census tract information used a prediction model including only race/ethnicity, while patients missing only census tract information used a prediction model with race/ethnicity and language.
