# Supplementary material for: Development and validation of a prediction algorithm to identify birth in countries with high tuberculosis incidence in two large California health systems
Source: PLoS One. 2022 Aug 25;17(8):e0273363. doi: 10.1371/journal.pone.0273363 (PMC9409495; doi:10.1371/journal.pone.0273363)
Supplement: S6 Table — (DOCX) [file pone.0273363.s007.docx]

**S6 Table: Screening Metrics for LTBI and TB in Patients with Unknown Country of Birth for Final Model Using Various Cut-points**

| **Model Combination** | **N Screened** | **True Cases Identified** | **True Positive Rate** | **NNS** |
| --- | --- | --- | --- | --- |
| **LTBI (applied to population screened for LTBI, N=1,393,704)** | | | |  |
| Preferred Language Only | 124,308 | 16423 | 0.21 | 7.6 |
| Final Model, 19% Cut-point | 309,828 | 37794 | 0.47 | 8.2 |
| Final Model, 24% Cut-point | 413,732 | 46090 | 0.58 | 9.0 |
| Final Model, 27% Cut-point | 454,637 | 48223 | 0.60 | 9.4 |
| Final Model, 34% Cut-point | 545,594 | 52645 | 0.66 | 10.4 |
| **TB (applied to full population, N=8,276,808)** | | |  |  |
| Preferred Language Only | 893,413 | 160 | 0.21 | 5583.8 |
| Final Model, 19% Cut-point | 1,734,125 | 462 | 0.62 | 3753.5 |
| Final Model, 24% Cut-point | 2,471,134 | 557 | 0.74 | 4436.5 |
| Final Model, 27% Cut-point | 2,747,142 | 571 | 0.76 | 4811.1 |
| Final Model, 34% Cut-point | 3,320,672 | 603 | 0.81 | 5506.9 |
