# Supplementary material for: Development and validation of a prediction algorithm to identify birth in countries with high tuberculosis incidence in two large California health systems
Source: PLoS One. 2022 Aug 25;17(8):e0273363. doi: 10.1371/journal.pone.0273363 (PMC9409495; doi:10.1371/journal.pone.0273363)
Supplement: S1 Appendix — (DOCX) [file pone.0273363.s008.docx]

**S1 Appendix**

Detailed definitions for each considered predictor, along with pros and cons for considering each from a clinical and subject matter expert perspective are provided below.

| **Predictor** | **Detailed Definition** | **Pros** | **Cons** | **Importance** |
| --- | --- | --- | --- | --- |
| **Preferred Language spoken in a country with high TB incidence** | Yes/No. Yes if patient indicates he/she prefers to speak a language spoken outside of Western Europe, Australia, New Zealand, or Canada. Languages spoken in countries both in and out of this region were assigned the category with the highest prevalence. For example, Spanish language preference was assigned as a speaking a language from a country of high TB incidence as more immigrants from California are from Mexico and Central America then from Spain. | High positive predictive value for those preferring to speak a language spoken in a high incidence TB country. | Missing patients who prefer to speak English | High |
| **Needs interpreter** | Yes/No, whether the patient has indicated he/she needs an interpreter for office visits | High positive predictive value for similar reasons as spoken language above | Cannot distinguish reasons for interpreter need - could be for languages spoken outside high incidence TB countries, even American Sign Language | Medium |
| **Race/Ethnicity** | Categorical variable for patient’s self-reported race/ethnicity (non-Hispanic white, non-Hispanic black, Hispanic, Asian, Hawaiian/Pacific Islander, Native American/Alaskan, or Other/Multiple/Unknown) | Literature has found strong correlation with race/ethnicity and positive TB status | Unclear relationship of country of birth to other, multiple, and unknown race/ethnicities. | High |
| **Past receipt of Bacillus Calmette–Guérin (“BCG”) vaccination** | Yes/No. BCG is a vaccine for TB disease. Many foreign-born persons have been BCG-vaccinated. BCG is used in many countries with a high prevalence of TB to prevent childhood tuberculous meningitis and miliary disease. | Very rarely indicated for US-born persons, so receipt is a strong indicator of birth in high TB incidence country | Very under captured in the EHR | Low |
| **Hepatitis B (HBV)Screening** | Yes/No. Whether patient has ever been screened at KP for HBV. | HBV is also a disease with disproportionate burden on those born outside the US, and there is overlap between high incidence TB and HBV countries | There are other indications for HBV screening which do not apply to LTBI screening | Low |
| **Percent Foreign Born in Patient's Residential Census Tract** | Continuous percentage. | Non-US-born individuals may have a higher probability of living near each other. | Includes countries outside high incidence TB countries, though in CA the overlap will be high | Medium |
