# Supplementary material for: Development and validation of a prediction algorithm to identify birth in countries with high tuberculosis incidence in two large California health systems
Source: PLoS One. 2022 Aug 25;17(8):e0273363. doi: 10.1371/journal.pone.0273363 (PMC9409495; doi:10.1371/journal.pone.0273363)
Supplement: S2 Appendix — (DOCX) [file pone.0273363.s009.docx]

**S2 Appendix: Description of model building process**

Starting from the main effects model, clinicians indicated BCG vaccination capture in the EHR was incomplete, and that indications for hepatitis B and LTBI screening criteria may not fully overlap. Additionally, the screening rate for hepatitis B was highly different in the population missing country of birth in the EHR, indicating this population may not have been adequately screened and that this variable would not extend to be an appropriate predictor. These variables were given low priority. Since additionally the AUCROC and AUPRC for these predictors were not satisfactory, these predictors were thus first removed with little change to model performance.

Needs interpreter was removed next as it was thought to measure something similar to language preference without the ability to separate out the languages spoken. Removal of this variable again resulted in a very small change in performance metrics.

The last step was to evaluate whether to include a model with preferred language, race/ethnicity, and percent non-US-born in census tract, or to just use a model with language and race-ethnicity (these two variables were given high importance and had the highest AUCROC and AUPRC in individual prediction models so they are to be included in any model). Removing percent non-US born in census tract resulted in a decrease in AUROC from .85 to .84 and in AUPRC from 0.70 to 0.68. These changes are still small, but because information on percent foreign born in a patient’s census tract maybe more valuable in cases where patient is missing information on race/ethnicity, which is more likely to be true in the population for which country of birth is missing (Table 1), we chose to include language preference, race/ethnicity, and percent foreign-born in residential census tract in the final model.
